# Slum decay in Sub-Saharan Africa: Context, environmental pollution challenges, and impact on dweller’s health

**DOI:** 10.1097/EE9.0000000000000158

**Published:** 2021-05-20

**Authors:** A. Kofi Amegah

**Affiliations:** Public Health Research Group, Department of Biomedical Sciences, University of Cape Coast, Cape Coast, Ghana

## Introduction

Slum settlements are a very prominent feature of the urban landscape of Sub-Saharan Africa (SSA) countries and other low- and middle-income countries (LMICs). SSA cities such as Accra, Ghana; Lagos, Nigeria; Nairobi, Kenya; Addis Ababa, Ethiopia; and Johannesburg, Cape Town and Durban, South Africa are home to some of the world’s largest slums due to massive urban growth in these countries over the past two decades. SSA records the highest number of slum dwellers. About 62% of the region’s urban population resides in slums compared with 35% in Southern Asia, 24% in Latin America and the Caribbean, and 13% in North Africa.^1^

Uncontrolled urbanization is the main driver of slum proliferation in LMICs. SSA and South Asia are the most rapidly urbanizing regions of the world with the highest population growth in urban areas found in these two developing regions. The rapid increase in urban population in SSA countries has been attributed to the high rate of natural increase in towns, reclassification of settlements into urban areas, and rural-urban migration, which is highlighted as the most significant driver.^2^ According to Teye,^2^ these factors will continue to drive the urbanization process in SSA countries. Africa’s urban population has been growing at a very high rate from about 27% in 1950 to 40% in 2015 and projected to reach 60% by 2050.

The United Nations Human Settlements Programme (UN-Habitat) defines a slum as an urban area with lack of basic services (sanitation, potable water, electricity), substandard housing, overcrowding, unhealthy and hazardous locations, insecure tenure, and social exclusion.^3^ Per this definition of a slum, it can be argued that such a settlement cannot experience any further decay.

In this commentary, I document manifestations of further decay in slums of SSA. The commentary answers three important questions; (1) can there be any further decay in slums of SSA? (2) how is the slum decay manifested? and (3) how is the slum decay impacting the health of dwellers? The article will have important policy implications in the region and beyond, and should help better tailor interventions for upgrading slums to improve, promote, and protect the health of populations living in these settlements.

## Manifestations of further decay in slums of SSA and associated environmental pollution problems

There has been a proliferation of unregulated industries in SSA slums in recent times especially food processing and sachet water producing industries. This is a result of cheap spaces in these settlements and low cost of labor. Diesel trucks and vans which are mostly old and rickety patronize the slums on a regular basis to cart goods from these industries to market and distribution centers. Emissions from these vehicles and the industries continue to worsen the already precarious ambient air pollution situation in the slums. The deepening *“*Okada” (commercial motorcycles) problem in West Africa in recent times is also very alive in slums. This is because most of the riders who are migrants reside and operates from the slums. The motorcycles are two-stroke engines and hence very polluting. A permanent feature of the slum landscape of SSA are old rickety mini buses (“trotro” as it is called in Ghana) and taxis which are also very polluting. Again just like the Okada, drivers of these vehicles reside and operate from the slums. This problem has, however, magnified in recent times owing to the growing population in the slums.

In many SSA countries, urban road development to ease traffic congestion in the inner-cities usually occurs at peripheries of the city and are the zones where most slums are located. Emissions from the vehicles plying these road networks also contribute to the high air pollution levels in these settlements.

Use of biomass fuel for cooking is universal in slums of SSA owing to endemic poverty and generates high levels of household air pollution in these settlements. Household PM2.5 levels from use of biomass fuels for cooking has been reported to be very high in Nairobi slums.^4^ Open burning of solid waste in households is also universal in slums due to the absence of waste collection services in these settlements and also contributes to the household air pollution problem. The haphazard and closely packed buildings in slums hampers waste collection agencies from extending household waste collection services to these settlements and deepening the open burning of waste at home. Skips strategically placed in slums for collection of waste can remain unattended for several weeks with decomposing waste from the overflowing skips also contributing to the ambient air pollution problem.

Commercial cooking ventures are dotted across the slum landscape of SSA countries. They have, however, increased in recent times owing to proliferation of industries in these settlements. Biomass fuel is the primary fuel of these ventures and contributes to the ambient air pollution problem. Roads in slums are unpaved with dust pollution, a common problem in these settlements and has magnified in recent times due to the growing expansion and extension of slums into adjoining areas. Dwellers in Nairobi slums perceive air pollution to be a major problem in their settlements^5^ and identify emissions from industries and dump sites as the main cause.^6^

In addition to the worsening air pollution problem in slum settlements of SSA, Water, Sanitation, and Hygiene (WASH) situation has also deteriorated. Open defecation has not been addressed in these areas. Pit latrines are being constructed close to surface water (streams, dams) and groundwater (borehole, unprotected wells) sources and have been documented^7^ to leach microbial and chemical contaminants including coliforms, *Escherichia coli*, fecal streptococci, ammonia, nitrates, and nitrites into these water resources. Endemic poverty in these slums hampers households from treating these unwholesome water resources before use. Choke drains and gutters are also a major problem in slums due to littering in these settlements and soil from the unpaved roads. Not until the COVID-19 pandemic, handwashing practices had not seen any improvement in slums of SSA due to the absence of running (pipe-borne) water in these settlements. The Coronavirus Disease 2019 (COVID-19) pandemic has, however, improved the situation with access to handwashing stations (veronica buckets and soap) increasing in households and public spaces especially. According to Amegah,^8^ it is important that these gains in handwashing habits are sustained after the COVID-19 pandemic to help reduce sanitation-related diseases in African countries.

## Context of slum decay in SSA

In SSA, almost all of the urban poor resides in slums.^9^ The poor are known to have practices that causes and deepens environmental pollution in the neighborhoods where they reside. These practices include use of biomass fuel for cooking and heating, rampant burning of garbage at home as a waste management strategy, open defecation, and littering among several others. According to Landrigan et al,^10^ household air pollution, unsafe water resources, and inadequate sanitation are some forms of environmental pollution that have been associated with poverty-related practices in low- and middle-income countries.

Ignorance is pervasive in SSA slums owing to high illiteracy levels of the dwellers and also, national civic education bodies are unable to reach these areas with their messages. Lack of electricity in slum settlements has also meant lack of access to information from watching TVs and listening to radio stations.

There is lack of governance structures in slums. Traditional African communities have chiefs, elders, and opinion leaders who maintain law and order, and discipline in their communities. Some communities also have Village Health Committees and Community Health Volunteers who are tasked with ensuring the health and welfare of community members. Good environmental conditions in communities is one of the many benefits derived from these governance structures in traditional African communities. They are also the avenue through which non-governmental organizations (NGOs), and local government and public health authorities can reach communities with information for improving their environment. The traditional communities also benefit from the services of community health workers who work in the communities. As part of their activities in the community, they are expected to educate community members on proper environmental sanitation. The absence of such structures in slums is part of the reason why these settlements are bedeviled with many environmental pollution problems.

Finally, there is an absence of communal spirit in slums. This is because slum dwellers are mostly migrants from different social and ethnic backgrounds, and as a result unable to forge a strong communal spirit necessary for mobilizing themselves for development or upgrading of their settlements.

## Environmental exposures and potential impact on health of slum dwellers

Poor people often live in areas where they have disproportionately heavy exposure to environmental pollution.^10,11^ Poor environmental conditions in slum settlements exposes dwellers to (1) pathogens including bacteria, virus, protozoa, and helminthes, (2) high levels of air pollutants including particulate matter, carbon monoxide, nitrogen dioxide, sulfur dioxide, and dioxins, (3) methane, hydrogen sulfide, and other noxious gases from decomposing waste materials, and (4) chemical contaminants including ammonia, nitrates, and nitrites that leached into ground and surface water from open drains and nearby pit latrines.

Environmental exposures in slums are a major health hazard and have been attributed to the high burden of disease and ill-health in these settlements. Gastrointestinal infections are highly prevalent in slums^12^ with children under 5 years of age, the most vulnerable.^13^ Choked gutters and drains serves as breeding sites for mosquitoes with dengue fever and malaria transmission levels very high in slum settlements. Aedes mosquito, the vector for dengue fever, for instance has been found to be adapted to slum conditions, in contrast to Anopheles mosquito, which thrives in high sunlight and plentiful vegetation.^14^

Cholera is frequently reported in slums especially during the rainy season and is compounded by the choked gutters and drains in these settlements. Typhoid transmission levels are very high in slums due to poor hygiene practices of food vendors. Systematic reviews of cholera outbreaks in Africa have identified slum settlements as the usual source of the epidemic.^15,16^

Asthma, rhinitis, allergies, and respiratory illnesses are also on the rise in slum settlements. According to Cooper et al,^17^ the unsanitary conditions in slums are associated with upregulation of inflammatory responses leading to a high prevalence of nonatopic asthma. This situation, according to the authors, is in sharp contrast to happenings in high-income countries where per the hygiene hypothesis, allergy results from excessive cleanliness. In addition, both ambient and household air pollution which are worsening in slum settlements are well documented to be associated with these and several other respiratory diseases.^18,19^

## Conclusion

This commentary highlights manifestations of further decay in slums settlements of SSA countries. Stopping the decay for population health gains requires a package of interventions including Community-Led Total Sanitation (CLTS) to end open defecation; distribution of veronica buckets to develop and improve handwashing habits; drilling mechanized boreholes to provide access to wholesome water; addressing air pollution through private sector involvement in waste management, provision of access to clean cooking solutions, and regularization of industrial development in slums; and community mobilization for dredging of choked gutters and drains, and paving of slum roads. In addition, strong advocacy for behavioral changes to improve environmental quality is needed in slums and should be led by local government and public health authorities.

## Conflicts of interest statement

The author declares that he has no conflicts of interest with regard to the content of this report.

## References

[1] United Nations Human Settlements Programme. The State of African Cities 2014: Re-Imagining Sustainable Urban Transitions. Nairobi: United Nations Human Settlements Program; 2014.

[2] Teye J. Urbanization and migration in Africa. United Nations expert group meeting for the review and appraisal of the programme of action of the international conference on population and development and its contribution to the follow-up and review of the 2030 agenda for sustainable development. New York: Population Division, Department of Economic and Social Affairs, UN; 2018.

[3] United Nations Human Settlements Programme. The Challenge of Slums: Global Report on Human Settlements. Earthscan Publications; 2003.

[4] Muindi K Kimani-Murage E Egondi T Rocklov J Ng N. Household air pollution: sources and exposure levels to fine particulate matter in Nairobi slums. Toxics. 2016; 4:E12. 29051417 10.3390/toxics4030012 PMC5606663

[5] Egondi T Kyobutungi C Ng N. Community perceptions of air pollution and related health risks in Nairobi slums. Int J Environ Res Public Health. 2013; 10:4851–4868. 24157509 10.3390/ijerph10104851 PMC3823347

[6] Muindi K Egondi T Kimani-Murage E Rocklov J Ng N. “We are used to this”: a qualitative assessment of the perceptions of and attitudes towards air pollution amongst slum residents in Nairobi. BMC Public Health. 2014; 14:226. 24597487 10.1186/1471-2458-14-226 PMC3973865

[7] Graham JP Polizzotto ML. Pit latrines and their impacts on groundwater quality: a systematic review. Environ Health Perspect. 2013; 121:521–530. 23518813 10.1289/ehp.1206028 PMC3673197

[8] Amegah AK. Improving handwashing habits and household air quality in Africa after COVID-19. Lancet Glob Health. 2020; 8:e1110–e1111. 32827474 10.1016/S2214-109X(20)30353-3 PMC7438063

[9] Ramin B. Slums, climate change and human health in sub-Saharan Africa. Bull World Health Organ. 2009; 87:886. 20454473 10.2471/BLT.09.073445 PMC2789375

[10] Landrigan PJ Fuller R Acosta NJR. The Lancet Commission on pollution and health. Lancet. 2018; 391:462–512. 29056410 10.1016/S0140-6736(17)32345-0

[11] Kjellstrom T Friel S Dixon J. Urban environmental health hazards and health equity. J Urban Health. 2007; 84(3 suppl):i86–i97. 17450427 10.1007/s11524-007-9171-9 PMC1891648

[12] Harpham T Stephens C. Urbanization and health in developing countries. World Health Stat Q. 1991; 44:62–69. 1926894

[13] Ezeh A Oyebode O Satterthwaite D. The history, geography, and sociology of slums and the health problems of people who live in slums. Lancet. 2017; 389:547–558. 27760703 10.1016/S0140-6736(16)31650-6

[14] Eisenstein M. Disease: poverty and pathogens. Nature. 2016; 531:61–63. 10.1038/531S61a 26981732

[15] Rebaudet S Sudre B Faucher B Piarroux R. Cholera in coastal Africa: a systematic review of its heterogeneous environmental determinants. J Infect Dis. 2013; 208(suppl 1):S98–106. 24101653 10.1093/infdis/jit202

[16] Rebaudet S Sudre B Faucher B Piarroux R. Environmental determinants of cholera outbreaks in inland Africa: a systematic review of main transmission foci and propagation routes. J Infect Dis. 2013; 208:46–54. 10.1093/infdis/jit195 24101645

[17] Cooper PJ Rodrigues LC Barreto ML. Influence of poverty and infection on asthma in Latin America. Curr Opin Allergy Clin Immunol. 2012; 12:171–178. 22391754 10.1097/ACI.0b013e3283510967 PMC7612855

[18] Requia WJ Adams MD Arain A Papatheodorou S Koutrakis P Mahmoud M. Global association of air pollution and cardiorespiratory diseases: a systematic review, meta-analysis, and investigation of modifier variables. Am J Public Health. 2018; 108(S2):S123–S130. 29072932 10.2105/AJPH.2017.303839 PMC5922189

[19] Lee KK Bing R Kiang J. Adverse health effects associated with household air pollution: a systematic review, meta-analysis, and burden estimation study. Lancet Glob Health. 2020; 8:e1427–e1434. 33069303 10.1016/S2214-109X(20)30343-0 PMC7564377

